# Pharmacogenomics and adverse drug reactions in children

**DOI:** 10.3389/fgene.2014.00078

**Published:** 2014-04-16

**Authors:** Michael J. Rieder, Bruce Carleton

**Affiliations:** ^1^CIHR-GSK Chair in Paediatric Clinical Pharmacology, Schulich School of Medicine & Dentistry, Robart Research Institute, Western UniversityLondon, ON, Canada; ^2^Pharmaceutical Outcomes Programme, Department of Pediatrics, University of British Columbia, BC Children's HospitalVancouver, BC, Canada

**Keywords:** children, pharmacogenomics, adverse drug reactions, ethics of research in children, childhood cancer

## Abstract

Adverse drug reactions are a common and important complication of drug therapy in children. Over the past decade it has become increasingly apparent that genetically controlled variations in drug disposition and response are important determinants of adverse events for many important adverse events associated with drug therapy in children. While this research has been difficult to conduct over the past decade technical and ethical evolution has greatly facilitated the ability of investigators to conduct pharmacogenomic studies in children. Some of this research has already resulted in changes in public policy and clinical practice, for example in the case of codeine use by mothers and children. It is likely that the use of pharmacogenomics to enhance drug safety will first be realized among selected groups of children with high rates of drug use such as children with cancer, but it also likely that this research will be extended to other groups of children who have high rates of drug utilization and as well as providing insights into the mechanisms and pathophysiology of adverse drug reactions in children.

## Genetics and childhood disease

The recent interest in personalized medicine has highlighted the importance of genetic variability in the pathophysiology and outcome of human disease (Weiss et al., 2008; Godman et al., 2013). In the case of children, the influence of genetics on health has been appreciated for many years; for example impact of congenital disease such as Down Syndrome was well known in the nineteenth century—although the precise genetic basis of the disease was not described until 1959 (Down, 1866; Lejeune et al., 1959). There are numerous other examples of genetically determined disease—such as Cystic Fibrosis and Duchenne Muscular Dystrophy—whose substantial impact on children's health, well-being and life expectancy have been known to pediatricians for many decades.

With respect to treatment, although many of the therapies that we are familiar with have only been used fairly recently—since the Therapeutic Revolution initiated by the discovery of the antibacterial activity of sulfanilamide in 1937—the potential that genetic factors could influence therapeutic outcomes was in fact recognized before that (Weinshilboum, 1987). Sir Archibald Garrod is recognized as the father of medical genetics by virtue of being the first investigator to recognize the potential importance of the fact that people are biochemically unique individuals. He also made the observation at the start of the last century that, in addition to controlling key metabolic pathways, genetics was also likely to influence variability in the response to patients to drugs (Knox, 1958).

This was confirmed in one of the landmark studies in pharmacogenetics, that being the research reported by David Price Evans, Victor McKusick and colleagues at Johns Hopkins who in their landmark study of isoniazid metabolism established that “fast” and “slow” acetylator phenotypes were the result of genetic variation between these two populations (Evans et al., 1960). In this paper, of the 291 subjects studied 82 were under the age of 20, of whom 57 were under the age of 12 (Evans et al., 1960). This paper also made the observation that the differences observed were the result of genetic variations that were not age-dependent, suggesting that what was important for adults would also be important for children.

While this work and the observations of others provided clear and compelling reasons that research toward understanding how genetics influenced drug action and clearance would be an important part of appreciating how to address variability in drug response—both desired and undesired—in children, pharmacogenomics has been a relative late comer to the forefront of pediatric research. It is likely that this was due to several factors, some of which interact. One of the major driving forces for pediatric pharmacology research—and, indeed for research involving children in other disciplines—has been the appreciation that ontogeny is an important element in the pathophysiology of many childhood disorders (Choonara and Rieder, 2002; Kearns et al., 2003; Hines, 2008; Dotta and Chukhlantseva, 2012; Kerkhof and Hokken-Koelega, 2012; Sävendahl, 2012). It has been appreciated for some time that an important determinant of drug response in children is in fact developmentally-induced changes in drug disposition related to maturation of pathways involved in drug activation and clearance, with the more recent appreciation that the ontogeny of drug receptors and transporters may be key elements in determining drug response in children (Kearns et al., 2003; Neville et al., 2011; Dotta and Chukhlantseva, 2012). As an example, in the fetus CYP3A7 is an important driver of oxidative drug metabolism while there is very little CYP3A4 activity; after birth, the amount of CYP3A7 rapidly declines while the activity of CYP3A4 steadily increases (Kearns et al., 2003; Dotta and Chukhlantseva, 2012). Renal drug excretion in the fetus is markedly reduced compared to older children; at birth term infants have a third of the capacity for glomerular filtration on a surface-area corrected basis compared to children at a year of age (Dotta and Chukhlantseva, 2012). Failure to appreciate this has lead to tragedy—for example, the Gray Baby Syndroe associated with chloramphenicol treatment in neonates (Choonara and Rieder, 2002). It is has been appreciated for several decades that developmental determined variability in drug clearance and response have had a major impact on drug safety in children (Choonara and Rieder, 2002). Thus, it is not surprising that much of the research conducted in pediatric pharmacology over the past several decades has focused on understanding the ontogeny of key determinants of drug activation, clearance response (Sumpter and Anderson, 2009). As well, most of the genetic disorders historically most relevant to child health were either chromosomal polysomies (for example, Down Syndrome) or disorders that are inherited by classical Mendelian or X-linked inheritance (for example, Cystic Fibrosis or Duchenne Muscular Dystrophy); in constrast, many pharmacogentically determined variations are due to markedly more complex mechanisms as well as frequently involving the interaction of several genes and gene products.

In addition to the issue of focus, there have been pragmatic issues that have constrained investigation in pharmacogenomics in children. The amount of blood needed to conduct studies—and the cost required to then analyze this blood—have been practical barriers that have historically constrained investigation in this area, notably among young children in whom there are technical difficulties in obtaining blood and limitations in terms of the volume of blood that can be drawn. In addition to the technical issues, the conduct of pharmacogenomic studies in children—indeed, the conduct of any research in children—involves the ethical consideration that the person consenting is not the person on whom the study is being conducted, and thus issues such as minimal risk and benefit/risk assume a different perspective in this population (Norbert and Roses, 2003; Avard et al., 2009). Finally, a barrier to drug research in children in general has been the perception—in fact, the myth—that drug use in children is both infrequent and is largely confined to antibiotics (Rieder et al., 2011).

While this challenges have been formidable, over the past decade many of them have been addressed and in fact there are now a number of pharmacogenomic studies in children underway (Stevens et al., 2013). The amount of sample needed for studies has decreased dramatically, while there has been an even more dramatic decrease in the cost and availability of genetic analysis (Loo et al., 2010; Hawcutt et al., 2013). Additionally, the availability of alternate sample sources for genetic studies—such as saliva—has greatly facilitated genetic studies in populations such as infants and toddlers (Loo et al., 2010). There has been emerging work on developing consensus in the approach to pharmacogenomic studies in children; recent work by our group has demonstrated that parents in fact are supportive of pharmacogenomic studies in children, with the proviso that this support was dependent on parental understanding of the reason(s) for the study and the potential benefit to their child and other children (Zhang et al., in press). Finally, it is now appreciated that in fact drug therapy in children is both common and complex—as an example, a Canadian child is prescribed on average 4 prescriptions a year from a range of more than 2400 therapeutic entities, while the use of psychotropic medications among adolescents in the United States has been one of the fastest growing trends in drug utilization over the past decade (Khaled et al., 2003; Rieder et al., 2003; Jonas et al., 2013). For some children—notably those with chronic disease—complex and frequently multi-drug therapy constitutes one of the mainstays of treatment. Thus the imperative for understanding sources of variability in drug use among children—notably with respect to drug safety—is both immediate and crucial.

## Genetics and optimal drug therapy

The recent interest in understanding how genetics controls drug disposition and response in children in turn leads to the consideration of how this new knowledge can best be applied to improve the care of children. There are a number of possibilities. Can insights into the genetic regulation of drug response lead to the identification of new therapeutic targets or to the more precise manipulation of known targets? Can the knowledge as to how genetics controls human drug variability be used to direct therapy to specific conditions in which a better response is probable? Can understanding genetic regulation of response and disposition of drugs in children be used to improve drug safety by better understanding how adverse drug reactions develop, how these reactions can be modified or prevented and which children are at specific risk for adverse drug reactions? While all three approaches should and are being pursued, it is likely that the most immediately fruitful results will come from applying pharmacogenomics to enhancing drug safety (Becker and Leeder, 2010; Hawcutt et al., 2013; Stevens et al., 2013).

## Adverse drug reactions in children

Pursuit of initiatives to improve drug safety in children argues that this is a pressing and important issue (Choonara and Rieder, 2002). While the Therapeutic Revolution and the development of specific therapy have been of unquestionable value in enhancing the health and well-being of children and in providing clinicians with unprecedented opportunities to cure and control common child health problems, this has not been without a cost. In addition to economic costs—and drugs are the second or third most expensive element in health care in many developed countries—drug therapy is associated with the risk of adverse drug reactions. Adverse drug reactions have been defined as “a response to a drug which is noxious and unintended and which occurs at doses normally used in man for prophylaxis, diagnosis, or therapy of disease, or for the modification of physiologic function” (Edwards and Aronson, 2000; Nebeker et al., 2004). Adverse drug event is a more inclusive term which includes errors related to drug administration such as giving the wrong drug or wrong dose (Kaushal et al., 2001).

Historically adverse drug reactions in children—typically in the context of a therapeutic tragedy—have been and continue to be major shapers of public policy on drug regulation (Choonara and Rieder, 2002; Ciszkowski et al., 2009). As noted above, children do in fact receive a number of medications—in our previous work, we have shown that in a cohort of 1,000,000 children there are on average 4 prescriptions written per child per year (Khaled et al., 2003; Rieder et al., 2003). What needs to be appreciated is that the use of prescription drugs is in fact not evenly distributed—while 65% of children receive either no or one prescription per year, 25% of children account for 70% of drug use (Khaled et al., 2003). These children are frequently children with complex and/or chronic disorders such as cancer, epilepsy, or asthma, and often are treated with multiple medications. As can be appreciated, adverse drug reactions are likely to be much more common among these children, and a number of studies have demonstrated that there is a significant rate of adverse drug reactions among children (Lazarou et al., 1998; Impicciatore et al., 2001; Le et al., 2006; Kongkaew et al., 2008; Davies et al., 2009; Aagaard et al., 2010; Sotomayor et al., 2010; Rieder, 2012). It appears that there approximately a 5% risk of an adverse drug reaction with most drugs used for the care of children, and for some drugs—such as chemotherapeutic agents—this risk is appreciably higher (Rieder, 2012) (Table 1).

**Table 1:** **Risk factors for adverse drug reactions in children**.

**Historical risk factors**	**Currently accepted risk factors**
History of a previous ADR	History of a previous ADR
Extremes of age	Extremes of age
Impairment of drug clearance	Impairment of drug clearance
Polypharmacy	Polypharmacy
Female Gender	Female gender
	Higher drug dose
	Certain genetic polymorphisms

Modified from Rieder (2012).

It is important to appreciate that, while pharmacogenomics may have much to offer in enhancing drug safety, it is likely that these benefits will be largest among specific sub-sets of adverse drug reactions. Adverse drug reactions can be classified by a number of systems, but one of the most useful groups adverse drug reactions as Type A (Predictable or Pharmacological) or Type B (Unpredictable) (Table 2) (Rieder, 2012). While Type A adverse drug reactions are more common, they are frequently mild and often self-limited (Davies et al., 2009; Rieder, 2012). In contrast, while Type B adverse drug reactions are less common, they are often more severe and are more likely to result in serious morbidity or even mortality (Rieder, 2012). Given that Type A adverse drug reactions can often be predicted from the known pharmacology of the drug, it is likely that pharmacogenomics will be of more utility in helping to address the problem of Type B adverse drug reactions. One exception is codeine toxicity, which as discussed below is a concentration-dependent toxicity related to the drug's known CYP2D6 pharmacogenomic variability in populations. An important classification of adverse drug events that pharmacogenomics has little relevance for are drug errors, which are due to individual and system issues in drug prescribing, dispensing and administration (Nebeker et al., 2004). In the case of drug errors, educational and system approaches are most likely to be fruitful in reducing the risk of these adverse drug events (Rieder, 2012).

**Table 2:** **Classification of adverse drug reactions**.

**Type A**	**Type B**
Predictable	Unpredictable
Common	Infrequent
Often mild, self-limited	Often severe
Based on the drug's known pharmacology	Based on genetic or other factors
Dose-dependent	No clear relationship to drug dose
Acts on drug's site of action or similar site	Unrelated to the site of drug action

Modified from Rieder (2012).

The importance of enhancing drug safety in children is even more telling given the drug approval process (Rieder, 2010). The usual submission for a investigational new drug does not include studies on children with the exception of drugs such as antibiotics, and thus many of the drugs prescribed for children are prescribed off-label, which is to say in populations and for indications that are not part of the marketing approval granted by a drug regulatory agency (Castro-Pastrana and Carleton, 2011; Kimland and Odlind, 2012). The paradox of this is that drugs which are shown to be safe and effective in specific populations—often adults—are then given to individual children, under circumstances in which the optimal dose and adverse effect profile may not be well defined (Castro-Pastrana and Carleton, 2011). By better understanding risk factors for adverse events—and potentially appreciating their mechanisms—pharmacogenomics offers the potential for greatly enhancing drug safety in children.

Historically there have been five recognized risk factors for adverse drug reactions, of which several—extremes of age and impairment of the organs of clearance—apply to children; more recently, an additional two risk factors have been added, one of which—certain genetic polymorphisms—is a direct indication of the importance of pharmacogenomics in drug safety (Table 1) (Rieder, 2012). Given the patterns of drug utilization in children, there are certain groups of children for whom pharmacogenomics is more likely to be of immediate benefit in improving drug safety.

## Childhood cancer

Cancer is one of the commonest causes of death for children in developed countries, but also has been one of the major—and often unsung—success stories of pediatric pharmacotherapy. Over the past five decades, there has been remarkable overall success in terms of improved survival among children with cancer (Armenian et al., 2013). However, the survival rates for some cancers—such as brain tumors—have not improved to the same degree, and over the past decade it has become increasingly clear that long-term adverse effects of therapy are impacting on the health and well-being of the survivors of childhood cancer (Rosoff, 2006; Armenian and Robinson, 2013). The rate of adverse drug reactions in children with cancer is high and many of the most dreaded complications of childhood cancer are adverse events directly related to drug therapy. These effects have significant immediate and long-term impacts as well as affecting health-related quality of life. (Fakhry et al., 2013). Interestingly, among survivors of childhood cancer, health-related quality of life is overall relatively high—except for those children who have sustained co-morbidities, typically as a long-term adverse events of therapy. In terms of frequency, this can be as many as two-thirds of all survivors; follow-up studies have demonstrated that a quarter of adult survivors of childhood cancer have serious chronic health conditions related to their therapy, with 25% having three or more chronic health problems (Rosoff, 2006). Given the complexity of cancer therapy and the fact that many of the drugs used are likely to be subject to genetically determined variability in disposition, childhood cancer would seem to be an area in which there is likely to be more rapid uptake of pharmacogenomics to enhance drug safety. Moreover, genetics is also a part of the care of some children with cancer, for example using the Philadelphia Chromosome (reciprocal translocation between chromosomes 9 and 22) to alter therapy—related to a different prognosis—in children with acute lymphoblastic leukemia (Bhojwani et al., 2009; Jabbour and Kantarjian, 2012).

**Figure 1:**
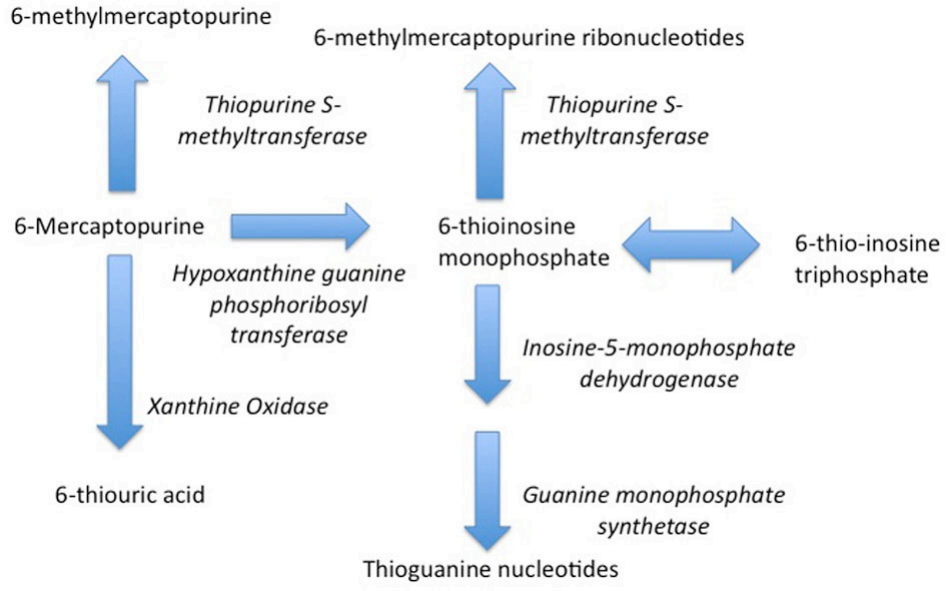
**Metabolism of 6-Mercaptopurine (6-MP)**. Metabolism of 6-MP via Hypoxanthine-guanine Phosphoribosyltransferase produces active Thioguanine nucleotide analogues while metabolism of 6-MP by Thiopurine Methyltransferase produces inactive 6-methylmercaptopurine. Genetically determined variations in 6-MP metabolism can produce significant differences in efficacy and safety.

Indeed, one of the first pharmacogenetic associations with drug safety—the pharmacogentic variability in S-methylation of thiopurines—was made in the setting of childhood cancer. Thiopurine Methyltransferase (TPMT) is a key enzyme in the biotransformation of 6-mercaptopurine (Figure 1) (Pacifici et al., 1991). 6-mercaptopurine is metabolized to a active metabolites—6-thioguanine nucleotides—mediated by hypoxanthine-guanine phosphoribosyltransferase (HGPRT) while methylation by TPMT produces active metabolites 6-methylmercaptopurine and 6-methylmercaptopurine ribonucleotides (Figure 1) (Weinshilboum et al., 1978). It has also been appreciated that this drug is associated with significant toxicity that is highly variable across populations (Weinshilboum and Raymond, 1997). A key study in this area was published in 1980, when Drs. Weinshilbaum and Sladek at the Mayo Clinic demonstrated marked variability in TPMT enzyme activity was related to autosomal codominant inheritance for alleles coding for low and high TPMT activity; of the subjects in the study, 88.6% had high enzyme activity, 11.1% had intermediate activity and 0.3% had no detectable activity (Weinshilboum and Sladek, 1980). Germane to the issue of drug safety in children, 115 of the subjects in this study were children, being on average 13 years of age. The clinical relevance of this finding relates to the fact that patients homogenous for low TPMT activity are at markedly greater risk for dose-related toxicity such as neutropenia during treatment with 6-mercaptopurine (Lennard et al., 1990).

The fact that this observation was made three decades ago raises the question of why TPMT genotyping has not been a part of routine care for children with cancer for longer than it has (McLeod et al., 2000; Bomgaars and McLeod, 2005). One pragmatic issue was that in some sense this discovery may have come too soon for implementation into routine care. At the time genotyping required significantly more blood—and more expense—than currently. The usual method of monitoring for neutropenia—doing a complete blood count—was a reliable and very inexpensive method of monitoring, notably as the initial considerations were this was only germane for the 0.3% of the population homozygous for low TPMT activity and as there were (and are) well established evidence-based protocols to monitor for this very complication. There has been active discussion and debate as to the extent to which dosage adjustment was needed in the much larger of patients heterozygous for the low activity allele (Relling et al., 1999). It has become increasingly apparent that dose alterations may be needed in other groups than the homozygous low activity group. As an example, patients with high activity may need an increased dose of 6-mercaptopurine, while the risk of a secondary malignancy may be related at least in part to variable TPMT activity (Bo et al., 1999; Adam de Beaumais and Jacqz-Aigrain, 2012). The role of gene-gene interactions in determining toxicity and the importance of a multi-factorial approach to risk assessment including consideration of age and concurrent therapy in addition to genotypic variability has highlighted the importance of genotyping, not only in the setting of cancer but also for other indications for thiopurines in children such as inflammatory bowel disease (Dorababu et al., 2012; Mazor et al., 2013).

More recently, pharmacogenomics has provided insights into other adverse events associated with therapy for children's cancer. Cisplatin is a platinum-based chemotherapeutic agent whose mechanism of action is to form *in vivo* cross-linkages to DNA in turn trigger apoptosis (Macciò and Madeddu, 2013). Cisplatin is a highly effective chemotherapeutic drug used for the treatment of a number of solid tumors in both adults and children. While a very useful chemotherapeutic agent, cisplatin produces many adverse events, one of the most serious being ototoxicity (Rybak et al., 2009; Brock et al., 2012). The known risk factors including concurrent therapy with other ototoxic drugs, male gender and age; particularly germane to child health care, children under the age of 5 have been found to have a 20 fold greater risk for ototoxicity than do adults (Langer et al., 2013). The mechanism of this ototoxicity—and which particular children are at risk—are not known although it is believed that oxidant stress may be a key determinant in this toxicity. Recently we conducted a study through a cross-Canada network of 16 pediatric academic health care center in cases of severe cisplatin-induced hearing loss and drug-matched controls were identified and studied (Ross et al., 2009). We found an association between tag single nucleotide polymorphisms (SNPs) in the *thiopurine S-methyltransferase* (TPMT) gene (rs12201199, rs1142345 and rs1800460) and in the *catechol-O-methyltransferase* (COMT) gene (rs9332377) with severe cisplatin induced ototoxicity (Ross et al., 2009; Pussegoda et al., 2013). This finding has been validated in a replication cohort but interestingly has not been validated in a murine model of cisplatin-induced ototoxicity (Pussegoda et al., 2013; Yang et al., 2013). We have modeled the predictive value of these alleles and our data suggest that children with no risk alleles have at the 5 year mark a 60% chance of having normal hearing, vs. children with three or more risk alleles who have a very high risk of having demonstrable hearing loss at the 5 year mark. Economic analysis has suggested that pharmacogenomic testing at the onset of chemotherapy can provide significant savings in health care costs (Dionne et al., 2012).

The approach taken to determine these SNPs should be noted. While one approach to studying pharmacogenomic sources of variability in drug disposition and toxicity it to use s study strategy tightly focused on pathways known to be mechanistically important in drug disposition and adverse events, we used a broader strategy given that the pathophysiology of cisplatin-induced hearing loss is not well understood. Consequently we studied 1949 SNPs in patients and controls to assess 220 genes involved in drug metabolism and disposition in addition to disease-specific genes that were related to pathways impacted by cisplatin therapy (Ross et al., 2009). This broad approach allowed us to not only potentially identify patients at risk but also to begin mechanistic studies to better understand how ototoxicity evolves and, potentially, how to prevent this.

We have used this approach to study the toxicity of another important class of chemotherapeutic agents, the anthracyclines. The anthracyclines a class of antitumour antibiotics; their mechanism of action involves intercalation between base pairs on DNA/RNA strands, inhibition of topoisomerase II, generation of iron-mediated free oxygen radicals and inducing histone eviction from chromatin (Gewirtz, 1999). The anthracyclines are extremely effective chemotherapeutic agents, and form the cornerstone of therapy for many common cancers in children, including most of the lymphotoreticular malignancies (van Dalen et al., 2011). In common with many potent chemotherapeutic agents, anthracyclines are associated with a number of serious adverse effects, of which the most feared long-term adverse event is cardiotoxicity (Kucharska et al., 2012; Harake et al., 2012; de Ville de Goyet et al., 2012). The known risk factors for anthracycline-induced cardiotoxicity include cumulative dose, female gender, higher dose rate, cranial irradiation and age. Similar to cisplatin, anthracyclines are associated with a markedly higher risk of toxicity in young children vs. adults (Harake et al., 2012; de Ville de Goyet et al., 2012). Anthracycline-induced cardiotoxicity is associated with significant morbidity and an appreciable mortality rate. Thus, identifying patients at risk would be of obvious advantage (Zerra et al., 2013).

Our group has studied this problem using a similar to that we used for our research with respect to cisplatin induced ototoxicity. Using a national network to identify cases of severe anthracycline-induced cardiotoxicity and drug-matched controls, we identified a series of SNPs associated with anthracyline-induced cardiotoxicity (Visscher et al., 2012). These variants include SNPs which appear to confer protection from anthracycline-induced cardiotoxicity such as loss-of-function SNPs for influx transporters which we believe bring anthracyclines into cardiac myocytes (Table 3) (Visscher et al., 2012). We have also identified risk variants which increase the risk of anthracycline-induced cardiotoxicity such as loss-of-function for efflux transporters which we believe export anthracyclines from cardiac myocytes (Table 3) (Visscher et al., 2012). We have replicated these findings in a cohort of children from the Netherlands and are currently validating these findings using cellular models (Visscher et al., 2013).

**Table 3:** **Risk and protective SNPs for anthracycline cardiotoxicity**.

**Gene**	**SNP**	**Predictive role**
*UGT1A6*	rs6759892	Risk
*ABCB4*	rs1149222	Risk
*ABCC1*	rs4148350	Risk
*HNMT*	rs17583889	Risk
*SCL28A3*	rs78583889	Protective
*FMO2*	rs2020870	Protective
*SPG7*	rs2019604	Protective
*SLC10A2*	rs9514091	Protective
*SLC28A3*	rs4877847	Protective

Derived from Visscher et al. (2012).

This research has yet to be translated into changes in clinical practice but points to the potential for pharmacogenomic work to enhance drug safety among children with cancer. Other drugs for whom pharmacogenomic studies are likely to be fruitful include cyclophosphamide and ifosfamide (Huang et al., 2000; Pinto et al., 2009; Johnson et al., 2013). Renal injury is a common and important long-term complication of ifosfamide therapy which is also much more common among children with cancer than among older patients. Our group has had extensive experience with this agent and we and others have demonstrated both that nephrotoxicity appears to be mediated by generation of reactive metabolites produced by pathways known to be subject to genetic polymorphisms and that N-acetylcysteine has potential as a preventive strategy to reduce the risk for and severity of ifosfamide-induced nephrotoxicity (Chen et al., 2008; Hanly et al., 2009, 2011). However, this is a resource-intensive and not entirely risk-free strategy, and thus pharmacogenomic approaches which could identity patients at risk would be of great use in the practical implementation of this approach to reducing an important toxicity associated with ifosfamide therapy, nephrotoxicity.

## Analgesic therapy

Over the past two decades it has been appreciated that historically pain has been under-treated in children and that this has been associated with poor outcomes (Taddio et al., 2010; Twycross, 2010). The WHO “Pain Ladder” describes a step-wise approach to pain management which includes the use of codeine, a weak opiate that has been one of the most commonly used opiates in children (Eddy et al., 1969; WHO, 1998). Codeine is the second most abundant alkaloid in opium and there are many reasons it has been commonly used for pain relief in children; it is inexpensive and is available orally in a stable formulation in both liquid and tablet form. Codeine itself is 3-methylmorphine and is a pro-drug. After codeine is taken, it enters the liver through the portal circulation where it is metabolized via both Phase I and Phase II pathways (Desmeules et al., 1991). Codeine de-methylated (mediated by CYP2D6) produces morphine, the major compound by which codeine exerts its analgesic effects (Sindrup and Brosen, 1995). Codeine conjugation by Glucuronyltransferase (specifically, UDP-Glucuronosyltransferase-2B7) produces codeine-3-glucuronide and codeine-6-glucuronide, 6-glucuronide being pharmacogically active (Madadi and Koren, 2008). Codeine metabolism by CYP2D6 typically only accounts for 5% of the dose, with further metabolism via glucuronidation producing primarily morphine-3-glucuronide and a much smaller amount morphine 6-glucuronide, the 6-glucuronide also being pharmacologically active.

Opiate toxicity, with the classical presentation of coma, miosis, and bradypnea, is a well-known adverse event seen with codeine overdose (Von Muhlendahl et al., 1976). This was felt to be very uncommon in usual therapeutic use. However, in 2006 Dr. Gideon Koren et al. described a therapeutic tragedy in which a breast-fed infant who of opiate toxicity related to maternal use of codeine post episiotomy (Koren et al., 2006). A detailed investigation of blood and breast milk coupled with genetic studies demonstrated that, despite the use of prescribed and conventional doses of a codeine-acetaminophen combination product by the mother, that the infant had very high concentrations of morphine in the blood, post-mortem blood morphine concentration being 245 nM/L (Koren et al., 2006). To put this in context, chronic high-dose opiate therapy produces blood concentrations in the range of 190 nM/L (Goucke et al., 1994). The reason for this very high morphine concentration became apparent when pharmacogenomic studies were conducted. The mother was an ultrarapid metabolizer for CYP2D6 who metabolized significantly more codeine to morphine than expected, validated by studies of morphine concentration in breast milk (Koren et al., 2006).

CYP2D6 has been recognized as a polymorphic enzyme for some time. There are three three distinct phenotypes—extensive metabolizers (EMs), poor metabolizer (PMs) and ultrarapid metabolizers (UMs) as the result of extensive polymorphisms for the gene encoding CYP2D6 (PheNeafsey et al., 2009). There are more than 80 recognized alleles for *CYP2D6*, with many of these producing the PM phenotype (Crews et al., 2012). These polymorphisms show variable expression in different populations (Bernard et al., 2006; Teh and Bertilsson, 2012). The importance of these polymorphisms in the metabolism of codeine was not appreciated initially. Codeine was first isolated in France in 1832, this being in a population among whom the UM polymorphism is uncommon. As the use of codeine has expanded it has become more common in populations with much higher rates of the UM genotype. Thus it is not surprising that problems with codeine toxicity have emerged—not only among newborns, but also among older children following surgery (Madadi et al., 2008; Ciszkowski et al., 2009; Kelly et al., 2012).

In contrast to the situation with respect to childhood cancer, these pharmacogenomic observations have resulted in a sharp response from drug regulatory agencies and a change in practice, with codeine use in children dropping sharply in many regions (Food and Drug Administration, 2012). Many hospital formularies have dropped codeine - sometimes with careful deliberation and sometimes with changes that less than fully thought out (for example, replacing codeine with oxycodone—which is O-demethylated by CYP2D6). A common change has been the replacement of codeine with morphine, probably sensible as most of the analgesic activity of codeine is related to metabolism to morphine (Poulsen et al., 1996). Of note, these changes occurred within five years of the publication of the index case that first triggered concerns as to genetically determined toxicity in children (Food and Drug Administration, 2012; Friedrichsdorf et al., 2013). It is worth considering why this happened so quickly with codeine as compared to the chemotherapeutic agents. In the case of chemotherapeutic agents, pharmacogenomic investigations have occurred in the context of children with severe disease with highly mortality for whom effective therapeutic regimens have dramatically changed outcome. Thus any change is likely to be carefully evaluated before changes in clinical practice occur. In contrast, while deaths in the context of codeine therapy have been infrequent they occurred in healthy children either after delivery or after procedures generally believed to be safe.

The case of codeine has highlighted the potential importance of pharmacogenomics for safe and effective analgesic therapy for children and how these variations can rapidly impact on public policy and clinical practice and suggests new directions for research in with respect to other analgesics and clinical practice implementation (Fukuda et al., 2013; Kelly et al., 2013).

## Drug hypersensitivity

One of the most feared patterns of adverse drug reactions among children is drug hypersensitivity such as Stevens-Johnson Syndrome and Toxic Epidermal Necrolysis (Elzagallaai et al., 2011). These are unpredictable, delayed onset, often very severe adverse events that occur not uncommonly and are associated with serious morbidity and mortality (Rieder, 2009). These adverse events occur with some of the drugs most commonly used for certain populations, such as carbamazepine, and to drugs which are being used more commonly such as sulfonamides (for example, for the therapy of community-acquired methicillin-resistant *Staphylococcus aureus*). Thus, developing strategies to evaluate risk at the onset of therapy would be of obvious utility and as well would provide important insights into the pathophysiology of these adverse events, which remains unclear (Rieder, 2012).

Initial pharmacogenomic studies in drug hypersensitivity have been somewhat disappointing (Rieder, 2012). Over the past decade the association has been made between drug hypersensitivity and certain haplotyes (Table 4) (Phillips et al., 2011). The use of these haplotypes to screen patients prior to initiating therapy has been proven as an effective strategy—most notably for abacavir, for whom the use of screening for HLA-B^*^5701 has reduced the risk of serious skin rash from 3% to zero (Phillips et al., 2011). However, this strategy also means that a number of patients for whom the drug would be effective and safe have been excluded from therapy—for example, in the case of carbmazepine as many as 6—8 patients who could have taken the drug vs. those who would have had a serious skin rash - suggesting that more detailed pharmacogenomic analysis would be helpful in more precisely identifying which patients would benefit and who would be at risk from therapy. As well, pharmacogenomic studies have the potential as noted above in helping to provide mechanistic insights that can direct both preventive and therapeutic strategies.

**Table 4:** **HLA Associations with adverse drug reactions**.

**Haplotype**	**Drug**	**Adverse event**	**Ethnic group**
HLA-B*1502	Carbamazepine	Drug hypersensitvity	Han Chinese
HLA-A*3101	Carbamazepine	Drug hypersensitvity	European
HLA-B*5701	Abacavir	Drug hypersensitvity	Mixed ancestry
HLA-B*5801	Allopurinol	Severe skin rash	Various populatons
HLA-B*3505	Nevirapine	Skin rash	Thai
HLA-B*5701	Flucloxacillin	Liver injury	Mixed ancestry
HLA-B*1502	Phenytoin	Drug hypersensitvity	Han Chinese, Thai

Derived from Phillips et al. (2011).

## Personalized medicine for children

The examples cited above should not be taken to mean they are the only care areas in which pharmacogenomics for children has the potential to improve drug safety. There are a number of other drugs commonly used in children for whom pharmacogenomic studies offer great potential in improving safety and efficacy (Downing et al., 2012; Lima et al., 2013; Vear et al., 2013). In some cases—for example, warfarin—pharmacogenomic studies in adults have already demonstrated the utility of incorporating pharmacogenomic data along with other variables in improving efficacy and reducing risk of adverse effects, albeit with some controversy (Siegel, 2012; Pirmohamed et al., 2013).

There is perhaps no area in which there is greater need for better defining therapy than pediatric psychopharmacology (Arango, 2011; Stroeh and Trivedi, 2012; Kimmel et al., 2013; Noam et al., 2013). While the use of psychoactive drugs in children has over the past two decades increased proportionately more than that of any other drug class, this increase in utilization has often occurred in the absence of robust evidence for efficacy, optimal dosage and safety. Given the large and increasing population of children receiving treatment for central nervous systems conditions—from epilepsy to depression—studies in defining optimal and safe therapy are urgently needed, and pharmacogenomics has considerable potential in not only defining efficacy and safety but also in asking key questions with respect to fundamental mechanisms underlying disorders involving the central nervous system in children vs. adults.

When considering these studies there are serious questions that need to be asked, such as which should be priority drug targets for study? This issue has been given careful thought by a number of investigators and algorithms have been developed to identify targets for further study (Shaw et al., 2013). While there are practical issues to undertake these studies, such as ensuring adequate numbers of patients, the development of national and international networks provide strategies that can address these issues (Loo et al., 2010). Future studies will need to include pharmacoeconomic evaluation as well as consideration of the evolving ethical climate under which clinical and genetic research is conducted (Wong et al., 2010; Donnan et al., 2011). In the case of pharmacoeconomic studies a critical element as health care systems globally struggle with the challenge of determining how to best allocate scant health care resources (Norbert and Roses, 2003; Hedgecoe, 2006; Rogausch et al., 2006; Fargher et al., 2007; Wong et al., 2010; Donnan et al., 2011; Pirmohamed, 2011).

## Ethical considerations

The use of pharmacogenomic information to inform diagnosis and guide therapy for children provides a series of interesting and unique ethical challenges. Implicit in therapy for children is the ethical conflict that the person giving consent—a parent or guardian—is not the one receiving the therapy. As an example, research conducting in the setting of childhood cancer demonstrated that when facing difficult therapeutic choices parents have difficulty in distinguishing their understanding of the situation from their child's experiences (Levi et al., 2000). This is further compounded by the controversy as to what constitutes assent and at which age assent can and should be obtained. This situation is very complicated in the United States, where the age of legal assent varies from state to state (Coleman and Rosoff, 2013).

A major additional challenge germane to pharmacogenomics is the concern that secondary information obtained during genetic testing may have a distant impact on access to insurance, employment or care (Netzer and Biller-Andorno, 2004). Again, this is an even more pressing issue when the person giving consent is not the person for whom the information is being obtained. An attempt to address this has been made with legislation such as Public Law 110–233 (110th Congress), the Genetic Information Non-discriminatory Act (GINA). This was signed into law in the United States Congress in 2008 with the intent of preventing insurance and employment discrimination on the basis of genetic testing (Dressler and Terry, 2009). While this was an honest and sincere effort to address this important issue with wide popular support—passing through the US Congress with only one dissenting vote—there are concerns that the rapid pace of development of genetic testing may render parts this legislation obsolete.

Our group has worked with parent groups in studying the ethical acceptability of genetic testing for their children and have found that their most important concerns were securing proper informed consent including ensuring that they had enough information to make a well informed decision. An additional concern was that their health care providers had the background and training to make appropriate decisions based on the results of genetic testing. This speaks strongly to the importance of patient and parent empowerment as well as to the need for including considerations on how to use genetic information for diagnosis and therapy in undergraduate and postgraduate health care professional training.

## Future directions

One of the major directions that medical education and care has made over the past several decades has been in the development of evidence-based medicine and in clinical guidelines informed by evidence to enhance and optimize patient care. Our group and others are working to incorporate the use of genetic information into guidelines on the diagnosis and therapy of common and important conditions. One area in which this is most likely to take fruit in the short term is the area of pediatric oncology, where guidelines are being written to incorporate the results of pharmacogenomic analysis into the initial assessment, monitoring, and therapy of children with cancer. Cancer and chronic disease offer the potential for pharmacogenomic analysis as therapy is being planned, which provides the opportunity given real-time turn-around of genetic testing to be able to better define which children are at special risk for adverse drug events and to better plan how therapy might be more closely monitored or modified in these children.

The potential of pharmacogenomics in improving therapy for children also applies in the development of new therapeutic agents, for which the potential for genetic variation impacting on efficacy and/or safety should be part of the research considerations and clinical trial design. Historically therapy for children has lagged behind therapy for adults—it is important that this should not be the case in the era of personalized medicine.

### Conflict of interest statement

The authors declare that the research was conducted in the absence of any commercial or financial relationships that could be construed as a potential conflict of interest.
